# Does Psychedelic Therapy Have a Transdiagnostic Action and Prophylactic Potential?

**DOI:** 10.3389/fpsyt.2021.661233

**Published:** 2021-07-19

**Authors:** Rita Kočárová, Jiří Horáček, Robin Carhart-Harris

**Affiliations:** ^1^Department of Translational Neuroscience, National Institute of Mental Health, Klecany, Czechia; ^2^Department of Psychology, Faculty of Arts, Charles University, Prague, Czechia; ^3^Beyond Psychedelics, Prague, Czechia; ^4^Department of Applied Neuroscience and Neuroimaging, National Institute of Mental Health, Klecany, Czechia; ^5^Third Faculty of Medicine, Charles University, Prague, Czechia; ^6^Centre for Psychedelic Research, Imperial College London, London, United Kingdom

**Keywords:** psychedelics, hallucinogens, psilocybin, psychological flexibility, plasticity, prevention, transdiagnostic, well-being

## Abstract

Addressing global mental health is a major 21st-century challenge. Current treatments have recognized limitations; in this context, new ones that are prophylactic and effective across diagnostic boundaries would represent a major advance. The view that there exists a core of transdiagnostic overlap between psychiatric disorders has re-emerged in recent years, and evidence that psychedelic therapy holds promise for a range of psychiatric disorders supports the position that it may be transdiagnostically effective. Here, we propose that psychedelic therapy's core, transdiagnostically relevant action lies in its ability to increase neuronal and mental plasticity, thus enhancing the potential for change, which we consider to be a key to its therapeutic benefits. Moreover, we suggest that enhanced plasticity *via* psychedelics, combined with a psychotherapeutic approach, can aid healthy adaptability and resilience, which are protective factors for long-term well-being. We present candidate neurological and psychological markers of this plasticity and link them with a predictive processing model of the action of psychedelics. We propose that a model of psychedelic-induced plasticity combined with an adequate therapeutic context has prophylactic and transdiagnostic potential, implying that it could have a broad, positive impact on public health.

## Introduction

To provide the background for our central thesis—that psychedelic therapy possesses a transdiagnostic and prophylactic potential—we first discuss current mental healthcare challenges before introducing a potential solution: the identification of a transdiagnostic treatment target that can aid prophylaxis or the long-term protection of mental health. We argue that psychedelic therapy has the potential to meet these two criteria. Key to our synthesis is evidence that psychedelics promote brain and mind “plasticity,” where plasticity can be defined in its purest sense as the quality of being easily shaped or molded (1). In the present paper, we use plasticity in this fundamental sense in relation to both the brain and mind (i.e., psychological phenomena), where enhanced plasticity of either phenomenon implies an enhanced capacity for change. In its purest, as well as in its classic biological sense, plasticity is closely related to “adaptability,” as the property of being easily shaped or molded naturally interfaces with conditional forces or factors that could shape or mold the plastic phenomenon in a particular way. In this paper, we aim to integrate the property of enhanced brain and mind plasticity *via* psychedelics with a predictive processing framework that is intended to apply equally well on psychological and neurobiological levels. This model constitutes an interdisciplinary approach to the mechanism of action of psychedelic therapy. Importantly, we explain how the model implies the prophylactic potential of psychedelic therapy—with implications for efforts to aid the development of psychedelic therapy into a regulated intervention for the recovery, promotion, and protection of psychological wellness.

Consistent with recent evidence supportive of these bold claims, we elect not to treat psychological health as a mechanistically discrete entity separate from pathology (2). In our view, reinforced “problematic” habits of brain and mind are universally relevant maladaptive processes (3). Put very simply, we argue that increased mental and neuronal plasticity, in combination with ideally supportive environmental contexts, can serve to promote psychological well-being on state and trait levels.

Mental health disorders are currently among the leading causes of disability worldwide. At least 300 million people suffer from depression, and close to 800,000 suicides are committed every year, with most linked to a mental health condition (4). There are thus, enormous human, social, and economic costs linked to mental illness (5), warranting that it be regarded as a major priority area.

Considering the magnitude of the burden of mental illness, its increasing global prevalence, and growing costs of healthcare (5), purely reactive interventions are unlikely to have sufficient impact (6). Moreover, there is a dearth of truly novel and effective new psychiatric drugs (7). Most current drugs have a lag in their therapeutic onset, possess a range of side effects (8), and show a modest efficacy relative to placebo (9–11); ~20% of patients are resistant to any of the presently licensed treatment options (10, 12). Moreover, pure pharmacotherapy, while convenient for “industrial” healthcare, may not have the correct action to target etiological causes in a manner that can serve long-term well-being (3).

While patients generally prefer talking therapies (13), questions remain over access, cost, speed of therapeutic action, and efficacy—particularly for severe cases (14). Thus, it is widely recognized that current treatments have limitations and that therapeutic breakthroughs are needed (5).

The World Health Organization (WHO) recognizes the potential value of proactive or preventative strategies for tackling the global burden of mental illness (5). Compared with preventative strategies in other domains of healthcare, progress in the prevention of mental illness has been poor (15). This may be due in part to industrial forces, including an excessive reliance on “myopic” (e.g., drug alone) interventions that may fail to target key underlying generative sources of illness (15). Consistent with this, a shift in focus in mental healthcare and research to strategies that promote wellness, and the capacity to address and cope adaptively with adversities seem justified and timely (15). The promotion of health and prevention of illness are interrelated in many respects (15)—a position backed by recent evidence of the continuous relationship between mental health and illness (2).

As the rapid discovery of effective vaccines for COVID-19 has demonstrated, an understanding of the etiology of a disease dramatically affects our ability to find effective treatments for it. In the context of mental illness, our incomplete understanding of the etiology of psychological suffering may be a major reason why our current treatments are not sufficiently effective. Regarding etiology, there is substantial evidence for an association between acute and chronic adversity and the development of many of the most prevalent and disabling psychiatric disorders (5, 16). Traumatic experiences during childhood are a well-known risk factor for various psychiatric disorders in adulthood (17). Decades of evidence has tended to suggest that the most prevalent psychiatric disorders (such as depression and anxiety disorders) feature a substantial environmental component in their etiology (3), albeit with a non-negligible but largely transdiagnostically shared genetic component (18) that seems to confer “vulnerability” or possibly even just “sensitivity to environment” — with direct and indirect links to plasticity (18, 19).

Thus, a large body of evidence implies the existence of individual differences in vulnerability to mental illness after adverse experiences (20). This differential sensitivity may depend on polygenic (18, 21), neurobiological (22, 23), environmental (24), psychological (25), and social factors (20). A composite vulnerability factor referred to as “resilience” has been the focus of research on preventative strategies in mental healthcare research (6), but is there a related and perhaps even more fundamental and tangible treatment target?

Two main strategies have been identified for the prevention of the development of mental health disorders and promotion of mental health: (1) the identification and potential mitigation of psychological, biological, community, economic, and environmental risk factors and (2) the strengthening of protective factors (26). We argue that psychedelic therapy can serve the latter factor in particular, by promoting a generalized mental and neural plasticity in combination with an ideally nurturing or supportive therapeutic context. Again, we adopt a standard dictionary definition of plasticity as the ability to change or be shaped by surrounding conditions (1).

Borrowing from genetic science (27), we also argue that a broad range of psychopathology can be conceived of as a maladaptive “canalization” of thought and behavior. Canalization can be defined as the maintenance of a trajectory (e.g., a style of thinking, feeling, relating, and/or behavior) that is resistant to change. Thus, in a sense, canalization is the inverse of plasticity. It is tempting to infer that canalized thought and behavior are an adaptive, defensive response to adversity that may thus, perhaps, paradoxically confer some (context dependent) evolutionary advantages (28). However, where such canalization creates maladaptive phenotypes—i.e., maladaptive in the context of a demanding modern western society—the logical way to treat it is to intervene to increase plasticity. Psychedelic therapy combines a plasticity-enhancing drug action with nurturing conditions—for the purpose of fostering a reset or recalibration of “maladaptive” habits of mind or behavior (29). Put most simply, we propose that the core therapeutic value of psychedelic therapy lies in its ability to open a window for healthy change.

There is a growing view that markers of mental health and treatment targets may be transdiagnostically relevant (30, 31). The transdiagnostic approach is characterized by identifying and targeting modifiable factors that traverse standard diagnostic categories (30, 31). This view is well-supported by the strong comorbidity of mental health disorders (32, 33) and the shared polygenic overlap between them (18).

A related perspective has inspired the Research Domain Criteria (RDoc) initiative in the USA, which places greater emphasis on pathologically relevant mechanisms rather than diagnostic categories, and has attracted major funding, if (as yet) limited breakthroughs (34). A similar direction is being taken by process-based psychotherapies, where mediators and moderators of mental illness and wellness are being explored (35, 36). If sufficient supportive evidence is gathered for either approach, it could have a far-reaching impact on treatment decisions as well as interdisciplinary bridging between different treatment models, orientations, and settings (35).

It would be fair to recognize psychoanalytic psychology here, as it has long recognized the existence of transdiagnostic overlap or commonalities between difference symptom-level expressions of psychological suffering, e.g., through the recognition of defense mechanisms (37). A greater alliance between psychoanalytic theory and practice and psychedelic therapy and science could be particularly fruitful going forward.

## Mental Health-Promoting and Health-Maintaining Effect of Psychedelic Therapy

**Figure 1:**
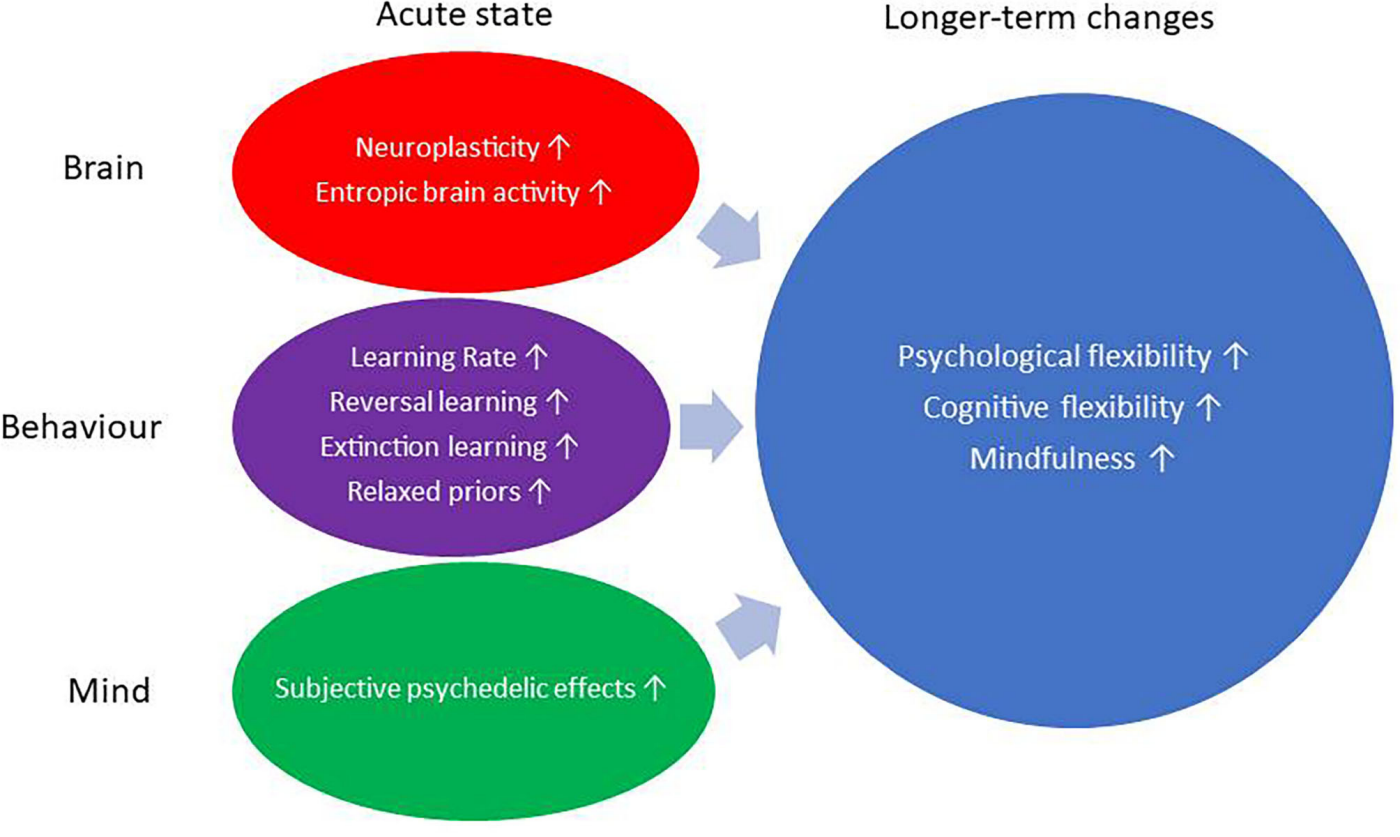
Mind and brain plasticity processes during acute state and follow-up.

Classic psychedelic (mind-manifesting) substances such as psilocybin (found in so-called “magic mushrooms”), dimethyltryptamine (DMT) (e.g., a key psychedelic ingredient in ayahuasca, a traditional psychedelic brew used in areas of South America), and lysergic acid diethylamide (LSD) have recently shown promise in the treatment of a variety of mental health disorders, including addiction (38, 39), obsessive compulsive disorder (40), depression (41–44), and existential distress in patients with life-threatening disease (45–48). Several ongoing clinical trials are exploring these and additional indications (49). There is also naturalistic and historical evidence supporting psychedelic therapy for functional neurological disorders (50), eating disorders (51, 52), psychosomatic disorders (53, 54), post-traumatic stress disorder (PTSD) (55–57), and perhaps even personality disorders (54, 58–61). This non-exhaustive list serves to highlight the potential transdiagnostic appeal of psychedelic therapy—implying that it may be able to address a core common denominator of mental health (see Figure 1). We propose here that this core component is a defensive canalization of thought and/or behavior vs. combining the plasticity promoting effects of psychedelics with their psychotherapeutic delivery in “psychedelic therapy” (28, 62).

Besides the treatment of mental health disorders, there is growing epidemiological evidence that psychedelic therapy can have positive effects on mental health in broader populations. The analysis of a representative sample of over 100,000 people in the USA showed that lifetime prevalence of psychedelic use was associated with a lower rate of past-year inpatient psychiatric treatment, prescription of psychotropic medication, and serious psychological distress (63, 64), as well as suicidal thinking, planning, and attempts (65). Psychedelic use was also associated with a lower rate of suicidal thoughts in a group of people with a history of childhood depressive episodes (63). Reduced rates of suicidality have now been shown in numerous naturalistic, observational studies involving a variety of classic psychedelics (66–68) as well as in controlled research with psilocybin (43, 44, 69) and ayahuasca for depression (70). One notable recent trial showed superior reductions in suicidality with psilocybin therapy vs. a 6-week treatment with a conventional antidepressant, albeit precorrection for multiple comparisons (44).

Naturalistic research into peyote (71) and ayahuasca (72) “users” revealed a better mental health profile in this population than in matched controls. A recent review of clinical trials and epidemiological studies (*N* = 77 eligible studies with 9,876 participants) documented that the use of psychedelics in various settings and populations was associated with aggregate improvements in a variety of indices of mental health (73). Other psychological changes linked to psychedelic use include prosocial attitudes and behavior (74), increased nature-relatedness (75, 76), and increases in the personality traits “self-transcendence” and “openness to experience” (77, 78). These ancillary domains are interesting and relevant to the topic of current paper; however, due to considerations of article length, we will focus mostly on mental health outcomes, albeit with a brief discussion of trait openness and creativity.

Taken together, the above-listed findings support the central hypothesis of this paper that psychedelic therapy can have transdiagnostically relevant, mental health-promoting effects. This proposition would be more compelling however, if plausible mechanisms were found that can account for these properties (22). We propose that the combination of plasticity-enhancing effects with a positive, supportive therapeutic context is conducive to psychological wellness across diagnostic boundaries, including one that would differentiate mentally healthy and unwell populations.

Mindful of projecting panacea-like properties into psychedelic therapy, we draw a specific line at mental illnesses with a clear organic etiology. We also recognize that there is a dearth of evidence to support the potential of psychedelic therapy to treat psychiatric disorders with a strong genetic contribution such as in the psychotic disorders. It has also long been suspected that psychedelic therapy may pose an elevated risk for iatrogenesis in individuals exhibiting at-risk factors for psychosis.

## Mental Plasticity and Psychedelics

There is growing evidence that psychedelic therapy promotes a variety of interrelated psychological traits (see Table 1) associated with better mental health outcomes and resilience to adversity, including psychological (79, 93) and cognitive flexibility (82, 103) and mindfulness (86–88) (see Table 1). The increase in these traits may be related to the quality of the acute subjective psychedelic experience, which includes such phenomena as “ego-dissolution” (104), near-death experience (105), emotional breakthrough (106), psychological insight (107), non-dual or unitive states (108), and other aspects of mystical or spiritual type experience (109). This list is not intended to be exhaustive but can rather serve as a heuristic for linking other potentially relevant psychological phenomena—which we do, to some extent, below.

**Table 1:** Table collating references to psychedelic or psychedelic-relevant studies showing increases in the relevant psychological constructs of processes of change.

**Psychological flexibility ↑**	**Cognitive flexibility ↑**	**Mindfulness capabilities ↑**	**Other relevant constructs ↑**	**Evidence for construct interrelations**
Davis et al. (43) Close et al. (79) Watts and Luoma (80) Zeifman et al. (under review) Belser et al. (81)	Murphy-Beiner and Soar (82) Boulougouris et al. (83) King et al. (84)	Murphy-Beiner and Soar (82) Domínquez-Clavé et al. (85) Sampedro et al. (86) Soler et al. (87) Uthaug et al. (88) Uthaug et al. (89)	**Resilience:** Brachman et al. (90) **Creativity:** Frecska et al. (91) **Insight:** Noorani et al. (92) **Connectedness:** Watts et al. (93) **Extinction and associative learning:** Catlow et al. (94) *Openness to experience:* Erritzoe et al. (95)	Levin et al. (96) McCracken and Velleman (97) Moore and Malinowski (98) Lebude et al. (99) Tomac (100) Coffey et al. (101) Biglan et al. (2008) Waugh et al. (102)

*Included in the “other relevant constructs” cell are “resilience,” “creativity,” “insight,” and different aspects of well-being and low-level associative learning (inc. extinction learning). These references are not intended to be exhaustive, and we reference just one for the “other relevant constructs” column to constrain the table's length. The rightmost column includes references to studies that have found correlations between the relevant constructs, supporting the view that they are indeed interrelated and therefore likely share common underlying mechanisms*.

We hypothesize that the processes of psychological change catalyzed by the pharmacological action of a psychedelic depend in large part on the triggering of plasticity. In a similar way to how the term “phenotypic plasticity” is used in evolutionary science, we use the term “mental plasticity” to refer to an individual's capacity to change his/her mind and/or behavior. Although this term is not established in the scientific literature, we believe it is a simple and useful term that is in-keeping with dictionary definitions and therefore makes no category error. We view mental plasticity as a psychological counterpart to brain plasticity or “neuroplasticity” — for which there are some well-established biomarkers. Many established and familiar markers of neuroplasticity are anatomical (110); but valid molecular (111) and functional markers (112) also exist. Some examples of mental plasticity and how they relate to mental health, and research with psychedelics, are discussed below. As highlighted above, for focus, we pay selective attention to psychological phenomenon linked to mental health.

### Psychological Flexibility

Psychological flexibility has been recently defined as the ability to feel and think with openness, to attend voluntarily to one's experience of the present moment, and to move one's life in directions that are important to him/her, building habits that allow him/her to live life in accordance with his/her values and aspirations [95, p. 5]. A six-component “Hexaflex” model has been conceived to describe processes relevant to psychological flexibility, and these include (1) acceptance (the inverse of experiential avoidance); (2) cognitive fusion (attaching thoughts to stimuli); (3) present moment awareness (closely related to “mindfulness”); (4) flexible perspective taking (self-as-context); (5) contact with values; and (6) committed action (113–115). The inverse of psychological flexibility, i.e., “psychological inflexibility,” has been described as a transdiagnostically relevant pathological phenomenon (116), associated with psychological distress (114), as well as with high cognitive and behavioral rigidity in depression (117), anxiety (118), and personality; substance use; and eating and obsessive-compulsive disorders (117)—all of which are candidate targets for psychedelic therapy (38, 40, 41, 47, 59, 119, 120). Cognitive fusion, the attachment of stereotyped thought to certain stimuli, is a key dimension of psychological inflexibility (121). Intuitively, cognitive fusion is mechanistically consistent with the phenomenon of canalization, i.e., in the psychological sense, the buttressed routing of certain styles of thinking and/or behaving, so that they become rigid and resistant to change.

Psychological inflexibility overlaps strongly with the related construct “experiential avoidance” (122). Indeed, like “emotional acceptance” (93), psychological flexibility refers to an individual's capacity for staying in contact with experiences eliciting negative emotions (i.e., emotional “acceptance”) as well as his/her commitment to living congruently with his/her own values (123). As indicated by this behavioral component, psychological flexibility is a focal construct for acceptance and commitment therapy (ACT), a “third wave” behavioral therapy (121, 124, 125), overlapping in theory and approach with mindfulness-based therapies (115).

It has been proposed that psychological flexibility and ACT are transdiagnostically relevant and conducive to long-term psychological wellness when promoted or practiced (121, 123). While not always an explicitly identified target, it has been proposed that psychological flexibility is a common component underlying several effective psychotherapeutic interventions (123). Increased interest in third-wave (126) and process-based (36) therapies, psychological flexibility (36), mindfulness (127), and contemplative practice in the West (128) parallels a rising interest in positive psychology (124)—reflective of common conceptual (129)—and mechanistic foundations.

Of the six “Hexaflex” processes, the acceptance component, defined as the “ability to allow and make space for all of one's internal experience, including thoughts, feelings, and sensations” (115), was identified as a key mediating factor in a qualitative analysis of patient–therapist interviews after participation in a clinical trial involving psilocybin therapy for treatment-resistant depression (TRD) (93). Increased experiential acceptance was also identified as salient in the ritual use of ayahuasca in the treatment of addictions (130) as well as in psilocybin therapy for end-of-life distress (81). A post-treatment increase in “connectedness” (131) and an expanded emotional range also emerged from a thematic analysis of 15 qualitative studies exploring patients' experience with psychedelics in the treatment of psychiatric disorder (107).

By supplementing these findings using the Brief Experiential Avoidance Questionnaire in a naturalistic, observational study, decreased experiential avoidance was found up to 1 month after psychedelic use in two large and separate populations (66). With the use of the Acceptance and Action Questionnaire-II (AAQ-II) in a separate sample but related design, consistent increases in overall psychological flexibility were seen (79); and in an independent retrospective survey study, psychological flexibility measured *via* the AAQ-II was found to mediate therapeutic changes after psychedelic experiences with various different compounds (132).

In summary, psychological flexibility is hypothesized to be a transdiagnostically relevant therapeutic phenomenon (116) linked to a specific therapeutic approach (i.e., ACT) that has been proposed to hold prophylactic value (121). It has also been proposed that ACT and the promotion of psychological flexibility in particular integrate well with psychedelic therapy (80, 115, 133, 134). Psychological flexibility also served as a guiding theme in a developing approach to psychedelic psychotherapy referred to as “Accept, Connect, Embody” or “ACE” (80).

### Cognitive Flexibility

Cognitive flexibility is a separate construct to psychological flexibility with a more experimental and translational heritage. It has been defined as “the ability to switch attention from one aspect of an object to another” (135). Cognitive flexibility is most often measured using behavioral paradigms that explore set-shifting and problem solving (136). Compared with psychological flexibility, cognitive flexibility is more closely linked with perseveration (its inverse) and is less overtly tied to psychopathology and therapeutic processes, although these connections have certainly been made (137). In the context of cognitive reappraisal strategies, individuals exhibiting high cognitive inflexibility struggle with switching attention away from internally focused negative rumination (138). Indeed, ruminative thought patterns in depression are closely related to—or indeed characterized by—cognitive inflexibility (138). There is evidence that cognitive flexibility is deficient in patients with eating disorders (139), general anxiety disorder (140), depression (117), autism (141), addiction (137), and obsessive-compulsive disorder (117) but may be enhanced by mindfulness-based treatments (140). There is also a link between positive mood and cognitive flexibility (142). It may be noteworthy that all of the above-listed disorders have been identified as viable indications for psychedelic therapy.

Increased cognitive flexibility has been observed 24 h after ayahuasca experience (82). Mixed findings were observed during the acute phase of LSD experience, with one study finding impaired cognitive flexibility (143) and another showing minimal effects on reversal learning (a construct closely associated with cognitive flexibility) in humans (144)—although some aspects of reward related learning were accelerated in this latter study, consistent with a clear, serotonin 2A receptor (5-HT2AR)-dependent acceleration of learning rate induced *via* LSD in rabbits (103). The 5-HT2AR is the key receptor site of action for psychedelics, where they function as agonists (145).

Reversal learning has been found to be enhanced by LSD in rodents (84). More evidence for an involvement of the 5-HT2AR in relation to reversal learning (146) includes the finding that antagonism of the 5-HT2AR promoted perseveration, reflective of cognitive *inflexibility* in rats (83) and rabbits (103). In humans, individuals with anorexia appear to exhibit both lower levels of 5-HT2AR expression (147–149) and lower cognitive flexibility than healthy controls (150). Several atypical antipsychotics possess 5-HT2AR antagonist properties (151); however, beyond some efficacy in parkinsonian (152) and dementia-related psychosis (153), there is little evidence that selective 5-HT2AR antagonists have appreciable efficacy for treating schizophrenia (154). That some effective antidepressant drugs have 5-HT2AR antagonist properties may be explained by their possession of a different (and in some ways inverse) therapeutic mechanism than is associated with psychedelic therapy, i.e., promoting an affective blunting conducive to symptom reduction vs. a psychotherapeutically mediated affective release conducive to symptom reduction plus ancillary benefits (44, 155).

Future work is needed to determine whether changes in cognitive flexibility with psychedelics are dose and context dependent. For example, one can imagine how cognitive fusion or perseveration (e.g., intrusive and repeating thoughts) could occur under psychedelics in a particular context, and this may be more likely during cognitive tasks that are improperly understood or difficult to implement due to a general, dose-dependent, non-specific (impairing) effect of the drug on cognitive function (156). Moreover, high doses might impair cognitive flexibility, while lower doses could conceivably enhance it.

So-called “divergent thinking” (157) and cognitive persistence (the persistent exploration of combinations of associations) have been identified as key factors constituting the creative process (158), and a link between cognitive flexibility, divergent thinking, and creativity has been made before (159–163). There is some empirical evidence (91, 164–166) and conceptual work (167–170) linking psychedelics with the promotion of aspects of the creative process. Nevertheless, creativity is a notoriously difficult phenomenon to satisfactorily define, and available research involving psychedelics is suggestive rather than confirmatory. Future studies may better dissect the interaction between psychedelics and creativity, as well as how this relates to mental health.

The personality trait “openness to experience” is relevant to the notion of mental plasticity (171). It has been shown to be positively correlated with divergent thinking and creativity (171), cognitive flexibility (172), and psychological flexibility (123). It was said by the founders of the Big Five assessment that open individuals “seek out novelty and variety and have a marked preference for complexity”; thus, open individuals are inclined toward curiosity and new experience (173).

Increased openness has been observed in clinical trials with LSD (174, 175), psilocybin therapy for TRD (95), and 3,4-methylenedioxymethamphetamine (MDMA)-assisted therapy for post-traumatic stress disorder (PTSD) (176). A relationship between acute “mystical-type experience” and subsequent increases in trait openness when it was assessed over 1 year after the psilocybin session has been observed (78). Although we consider openness to experience an interesting phenomenon in regard to the theme of mental plasticity, there is mixed evidence on its relationship with mental health (177). One possible direction for future studies may be to examine differential changes in the different subfactors of openness to experience (177).

### Mindfulness

Mindfulness has a heritage in Buddhist practice and philosophy (127). Mindfulness has been defined as the process of regulating attention with the aim of bringing its focus onto one's current experience, observing lived experience with curiosity, openness and acceptance, and also processing gained insights (178). A widely used five-facet operationalization sees mindfulness as the capacity to (1) observe, (2) describe, and (3) act with awareness of present moment experience, with a (4) non-judgmental and (5) non-reactive attitude (179). Mindfulness has been framed as a construct that overlaps with emotional regulation (101) and some processes of psychological flexibility (180).

Mindfulness has been widely studied and applied in diverse areas, and mindfulness-based techniques have been found to be useful in the treatment of mental illness and promotion and protection of psychological well-being (178). Mindfulness encourages the abandonment of ineffective experiential avoidance strategies and adoption of an adaptive response to stress (178). Mindfulness capacity has been shown to be positively correlated with psychological (96, 97) and cognitive flexibility (98) as well as creativity (99) and resilience (100).

Open-label observational studies have explored the effect of ayahuasca on mindfulness capacities as measured by the Five-Facet Mindfulness Questionnaire (FFMQ) (179). Significant increases in some mindfulness capacities were observed 24 h after ayahuasca, specifically in non-judgmental and non-reactive processing and observing (85) as well as acting with awareness (86–88). Relatedly, in a controlled study using natural language processing applied to participant responses during semi-structured interviews, participants on LSD were found to use fewer references to the past—suggestive of a more present-centered state. Moreover, this effect correlated negatively with default-mode network connectivity—a network related to daydreaming or “mental time-travel”—in some ways the opposite phenomenon to mindfulness (181). Another recent open-label observational study showed significant increases in mindfulness capacities after inhaling secretion from the parotoid gland of the *Bufo alvarius* toad, which contains the serotonergic psychedelic 5-methoxy-*N, N*-dimethyltryptamine (5-MeO-DMT) (89).

### The Acute Psychedelic Experience

There exists a large body of evidence to support the principle that the quality of an individual's acute experience under a psychedelic reliably predicts and mediates longer-term psychological outcomes—including changes in mental health outcomes (182–184) as well as other trait factors (78, 95, 185). There is also evidence to suggest that certain traits, such as absorption (186) and contextual framing or priming (109), can shape both the intensity (183) and nature of this experience (29, 109, 183). Perspectives differ on how best to refer to intense subjective experiences under psychedelics. Some prefer the more secular term “peak experience” — with its origins in Abraham Maslow's work (182)—while the term “mystical-type experience,” as measured through the “mystical experience questionnaire,” is preferred by others (38, 74, 109, 187).

A progressive step toward framework agnosticism might be to focus on the mechanisms underlying such experiences. In this regard, the term “unitive experience” has a good theoretical and empirical heritage (188, 189) as well as an intuitively mechanistic appeal (62, 190). Feelings of unity, universal interconnectedness, or oneness are closely related to the constructs of non-dual awareness (191) and ego-dissolution (104, 108, 192), which have developing empirical and neurobiological foundations (185, 193, 194) and have a heritage in Eastern philosophy and practice (195, 196).

Unitive or non-dual states of consciousness, marked by “ego-dissolution” and a sense of oneness or interconnectedness, sometimes framed as “peak” or “mystical-type” experiences, are reliably induced by psychedelics in a dose-dependent manner (104, 108, 109). As has been empirically shown, at the level of global brain function, one might expect to observe this effect reflected in a flattened energy or attractor landscape (197), consistent with a system that is both globally interconnected and desegregated (190) as well as more supple, pliable, and “free” in its functioning, i.e., being capable of visiting a broader repertoire of sub-states—rather than being confined to just a few dominant ones (198, 199). A range of recent neuroimaging studies with psychedelics have lent significant support to this characterization of brain function under psychedelics (190, 193, 194, 200–202).

In what follows, we review findings of increased neuronal plasticity *via* psychedelics and finally seek to integrate the relevant material into a comprehensive “process of change” model that places acute and (potentially) subacute increase in mind and brain plasticity at the core of a potentially long-term therapeutic change.

## Neuroplasticity

According to one definition, neuroplasticity is the ability of the nervous system to reorganize its structure, function, and connections in response to a changing environment or a shifting set of demands, thus constituting the mechanism of neuronal adaptability (203–205). In mental health disorders and certain neurological conditions (206), the (mal)adaptive strengthening of specific neural circuits can underlie dysfunctional patterns of thought, emotion, cognition, and behavior (204); moreover, functional organization can appear atypically modular, segregated, or inflexible in low mood states and in populations of people with depression (207, 208). Chronic stress has been found to disrupt neuroplasticity (203, 209) but may also promote it (155, 199). Some psychiatric treatments, e.g., antidepressant drugs (203), ketamine (210), and psychedelics in particular (110, 211–213), appear to promote neuroplasticity, albeit in inconsistent brain regions, e.g., the hippocampus with selective serotonin reuptake inhibitors (SSRIs) and in the cortex, in particular, with psychedelics.

Based on their ability to promote cortical neuroplasticity (110), a new name for psychedelics has been recently proposed: “psychoplastogens” (212). A variety of interventions for mental illness appear to promote neuroplasticity, including monoaminergic antidepressants (214), transcranial magnetic stimulation (204), and mindfulness meditation (215). Congruently, two independent studies have found that the intensity of psychotomimetic and dissociative symptoms experienced during ketamine-infusion positively correlated with its sustained antidepressant effects (210, 216, 217)—which is assumed to be mediated by the rapid induction of neuroplastic effects (210)—although see also Olson (218). The paradox of an acute “psychotomimesis” mediating a subsequent therapeutic change is relevant here, as it is to classic psychedelics (174).

Perhaps the most compelling evidence for increased neuroplasticity post-psychedelics derives from a recent study that found increased synaptogenesis with a range of different classic serotonergic psychedelics, ketamine and MDMA (110). Separately, a doubling of a marker of neuroplasticity, brain-derived neurotrophic factor (BDNF), was found in the cortex of rodents after exposure to a psychedelic in two separate studies (211, 219), but inconsistent results have been found in the hippocampus (94, 220). A recent gene expression study found clear evidence of increased plasticity gene expression in the cortex with psilocybin with some evidence for related effects in the hippocampus (213).

Constituents of ayahuasca have been linked to neurogenesis *in vitro* (although not *via* the main psychedelic constituent, DMT) (221), and a particular dose range of psilocybin has been found to promote extinction learning in mice (94) as well as a 5-HT2AR-dependent acceleration of association learning rates in rabbits (103). A further recent study found that proliferation of basal progenitor cells was under the control of 5-HT2AR stimulation and disproportionately so in humans relative to evolutionarily “lower” (i.e., by cortical mass) mammals (222), and other recent evidence has shown a role for 5-HT2AR signaling in neurite development (223).

As may be true of plasticity more broadly (224), neuroplasticity may be “outcome-agnostic” (19), in the sense that it could arguably just as easily mediate pathogenesis as salutogenesis or wellness—consistent with the so-called “plastic paradox” (205). This paradox relates to the essential role played by context in determining outcomes from psychedelic therapy (29) as well as recent conceptions of the role of serotonin in mediating mental health outcomes (225). See here for a thorough review of these themes (19).

### Entropic Brain

The network level changes described above may relate to the dysregulation of ongoing brain activity (194, 226) and an associated increase in entropy or complexity of spontaneous population level neural activity (185, 227–229)—as captured by the “entropic brain” hypothesis (228, 230). Based on the hypothesis that a broad range of psychiatric disorders feature inflexible brain dynamics, it has been proposed that psychedelics first dysregulate spontaneous brain activity and then allow it to reset or recalibrate in a less aberrant, more typical way (62, 119, 231). A related “reset” (119) or recalibration analogy for the therapeutic mechanisms of psychedelic therapy has recently been supported by brain imaging findings after psilocybin therapy for TRD (231). A period of emotional equanimity and well-being, referred to as an “after glow,” is often reported in the days–weeks following a psychedelic experience (86). Future work is required to better determine the neurobiological character of the relevant processes and phenomena here, including the putative reset/recalibration, as well as “after-glow” effects [but see (62)].

It is tempting to infer that the entropic effects observed acutely under psychedelics are reflective of a functional neuroplasticity that is a mediating vehicle for the relevant longer-term psychological changes that are the focus of this review [e.g., see (185)]. Psychedelics may hijack key neuronal adaptability mechanisms by directly activating them *via* the 5-HT2AR (62). Within this model, entropic spontaneous brain activity is just one level of a multilevel, generic brain and mind plasticity (229).

## Toward a Unified Process of Change Model

So far, we have reviewed relevant phenomena and observations of mind and brain plasticity linked to psychedelic use and therapy. We now attempt to synthesize these various phenomena into a multilevel “process-of-change” model. This model takes inspiration from the Bayesian brain or predictive-coding mechanisms, and particularly how it has been applied to the action of psychedelics (62). In brief, we propose that the main benefit of either the acute neurobiological or psychological effects of psychedelics is the opening of a window of plasticity for a therapeutic change. This can be accomplished through an appropriately supportive context, enabling the mind and brain to be recalibrated in a more adaptive and healthier way (19).

We begin by speculating that entropic brain effects of psychedelics (230) are related to increases in more established markers of neuroplasticity (110, 213, 219) as well as their core therapeutic effects. 5-HT2ARs are densely expressed in the cortex (232)—where increased plasticity has been most compellingly demonstrated post psychedelic (213, 220). One can imagine how entropic brain activity could service an accelerated learning rate (103), including accelerated extinction learning (94). It is easy to intuit that such conditions would be conducive to the relaxation and revision of canalized thoughts and behaviors linked to various psychiatric disorders. Previous work has framed such canalization and its sensitivity to psychedelic therapy in a predictive coding manner, where canalized thought and/or behavior would be related to excessive precision-weighting on “priors” (i.e., internal predictive models that encode implicit assumptions), and these become relaxed or “de-weighted” through the entropic action of psychedelics (62) in combination with therapy (19). While useful only as a metaphor, the last author has used a shaken “snow globe” analogy to refer to the entropic brain action of psychedelics and subsequent psychological recalibration process.

Setting some context for these ideas, the paradox of destabilization or dysregulation being a requirement for a learning process serving therapeutic growth is not entirely anomalous in medicine or cognitive and computer science (233). For example, periods of destabilization in the psychotherapeutic process have been found to predict better eventual outcomes (234), and a transient worsening of the symptoms can sometimes precede therapeutic breakthrough (235)—a process referred to historically as a “healing crisis” (236). It is logical that the process of breaking down canalized thought/affect/attitudes/behavior will evoke anxiety—but if appropriately prepared for and “surrendered to” (237), it may also be welcomed, e.g., as an opportunity for therapeutic growth or learning (106).

Psychological flexibility, and especially its component “cognitive fusion,” fits neatly within the above-described prediction coding model, where psychedelic therapy is proposed to relax over-weighted mental and neuronal habits or “priors” (62). In mental illness, certain defensive habits would be expected to have a gravitationally attracting influence, routing thought and behavior in a particular way. From a dynamical systems perspective, the notion of a “limit cycle” is relevant here (238). Examples of canalized thought or behavior in mental illness include ruminative thinking, obsessive thoughts, or compulsive behavioral patterns. Cognitive de-fusion nicely captures the mechanism of effective psychedelic therapy being outlined here. With proper psychological preparation, support, and integration, an individual's psychedelic process is guided toward the ideal of enhanced long-term psychological flexibility (80, 115), through the vehicle of an acute brain and mind plasticity aiding a healthy learning process. From a dynamical systems perspective, the image of a flattening energy landscape is relevant (200).

It is common for individuals who have had a psychedelic experience to report personal or transpersonal insights (107). Indeed, such insights are often regarded as being another major mediating vehicle for subsequent psychological transformation (62) and speak to the principle of psychedelics enhancing a learning process. Insight and emotional breakthrough are common features of psychedelic experiences and are recognized components of effective psychotherapy more generally (239). In the context of psychedelic therapy, their occurrence strongly predicts positive long-term clinical outcomes (69, 182). The opening-up of a new perspective may be a common consequence of an acute entropic brain effect (185)—and recent evidence of increased bottom-up information flow under psychedelics hints at relevant mechanisms by which insight may occur, namely, reduced top-down and increased bottom-up information flow (240).

Why do cognitive fusion, canalization, and the over-weighting of priors occur at all? It has been proposed elsewhere that the development of psychological rigidity is an adaptive response to psychological distress linked to actual or perceived adversity. In psychological terms, cognitive fusion may be conceived of as a defense mechanism (28), where its function is to counteract feelings of distress and uncertainty (28). Feelings of uncertainty and helplessness are, of course, a feature of early life (241), reflecting a critical period of brain development and neuroplasticity in which life experiences have an exaggerated impact on the developing mind and brain (242)—helping explain why early life adversity is such a strong predictor of later life psychopathology (16). Maladaptation to adversity is not an inevitability of course, e.g., as reflected by post-traumatic growth (243). It is telling in this context that greater scores of psychological flexibility are associated with lower scores of post-traumatic stress and higher scores of post-traumatic growth (244). See also Brouwer and Carhart-Harris (19).

Lastly, we have noted that brain regions showing the densest expression of 5-HT2AR (232) are also regions that show the longest-range cortico-cortical connections (245), the lightest myelination (246), and the highest intersubject variation in functional connectivity (245). These regions process longer temporal sequences of information (247) and more abstract semantic material (245, 248). They are most spatially removed regions from unimodal cortex (249, 250), show a consistent gene expression (251), have undergone greatest evolutionary expansion, from primate to human (246, 252), and show the greatest expansion throughout ontogenetic development.

These regions also feature more aerobic glycolysis (253) and amyloid deposition than elsewhere in the cortex (254). Recent findings suggest that cortical expansion may be under the control of 5-HT2AR signaling (222) and 5—that this signaling also plays an important role in neurite development (223). We highlight these interrelated properties as they imply an important role for the 5-HT2AR in activating and accelerating growth at various stages of life. Evidence linking 5-HT2AR signaling with the evolution of the human brain is particularly intriguing, raising questions about when and why a deepening of mind and brain plasticity could be advantageous to our species, as a transient state (e.g., the psychedelic state being a drug-induced hyperplastic state), a carryover effect of such states, or a more tonic quality of plasticity. See Brouwer and Carhart-Harris (19) for a relevant discussion.

## Discussion

This paper has reviewed literature supporting the claim that psychedelic therapy may have prophylactic or preventative potential in mental healthcare, and a related transdiagnostic therapeutic action, focused on increased brain and mind plasticity. We have supplemented these claims by presenting a process of change model that can be used as a template for investigating and understanding *how* psychedelic therapy possesses these valuable properties. More specifically, we have proposed that *via* an acute state of heightened mental and neuronal plasticity, flanked by psychological support designed to shepherd psychological growth or learning in a therapeutic direction, psychedelic therapy targets a core dimension of mental health that traverses diagnostic boundaries, including the one between health and illness. We have used a predictive processing framework to describe this “process-of-change” model, where acute plasticity combined with psychological support is utilized to promote a healthy re-learning, where what is re-learnt is an equanimous, open, and psychologically flexible state of being. This process could be described as a recalibration—of brain, mind, and behavior—serving current and long-term psychological health.

In terms of limitations, the present paper has not sought to critically appraise our central hypothesis that psychedelic therapy can aid prophylaxis or possess a transdiagnostic action. It can therefore be fairly criticized for cherry-picking findings supportive of its central narrative. We also acknowledge that while we have presented a process of change model, as yet few studies have been done that can directly connect the various model components in the same population, e.g., acute brain plasticity (in its various forms) with entropic brain activity and pre- vs. post-psychedelic changes in relevant psychological and neurobiological domains, such as psychological flexibility. The paper could also be criticized for being too cognitive-centric.

One relevant recent debate concerns the question of whether acute subjective effects are necessary for longer-term therapeutic outcomes, with one view being they may be useful but not essential (218) and another view favoring their essentialness (255). A related question concerns whether and by what magnitude does positive expectancy drive therapeutic outcomes. Positive expectancy is often wrongly referred to as the “placebo effect”—a term that should strictly be reserved for positive expectancy driving positive outcomes from an inert intervention. Our assumption is that positive expectancy is an important contributor to positive outcomes from psychedelic therapy [e.g., see (256)]. Moreover, it seems reasonable to consider it as a harnessable component of the model itself. Perhaps the utilization of positive expectancy could even be considered best practice, and studies that allow for it could be viewed as more ecologically valid than those that attempt to strip it out. Even if positive therapeutic outcomes were to be observed from a non-psychedelic “psychoplastogen,” one could not easily rule out a role for positive expectancy (212, 257). There may be a number of cautionary factors to consider if psychedelic therapy is to be legitimately explored as a preventative measure—perhaps foremost is the risk of iatrogenesis (258). While controlled and population studies indicate the prevalence of iatrogenic reactions is very rare, it remains plausible and not without precedent that a worsening of mental health after a psychedelic experience is possible (54)—particularly if the drug is used contrary to safety guidelines (259). The risk of hallucinogen-persisting perceptual disorder also needs to be considered, despite its prevalence appearing to be very low (260).

Like others (29, 261, 262), we have previously highlighted the importance of contextual factors (often referred to as “set and setting”) in mediating the outcome of a psychedelic experience, but there may be additional risk factors to consider. Psychological preparation, support, and integration are likely to be essential components of any future therapeutic and prophylactic application of psychedelics, and while some progress has been made in demonstrating the positive influence of preparation and support on subsequent outcomes (183), much more needs to be done, including measuring the role of psychological integration—in its various forms. Thus, based on the same mechanistic principle, in the same way that psychedelics may facilitate adaptive processes, if used improperly, they could potentially augment maladaptive processes, e.g., re-triggering trauma and/or associated defense mechanisms—which may manifest as over-weighted or “canalized” priors.

Relatedly, while the maladaptive canalization and associated “too strong priors” model appears to fit well with a broad range of psychiatric disorders and their main symptom clusters, it does not fit them all. For example, autism has been described as a condition in which weak high-level priors permit a hypersensitivity to sensory input/prediction error (263, 264); and certain psychotic states, such as delirium, or aspects of early psychosis seem more consistent with a weak prior model. To clarify, with some qualification, psychiatric symptoms consistent with the “too strong priors” model are those we believe to be most amenable to effective treatment *via* psychedelic therapy, as long there is an acceptably low risk of triggering a catastrophic psychological decompensation if these defensive priors are challenged.

From a dynamical systems theory perspective, it is hypothesized that psychedelics flatten the mind and brain's energy landscape, implying that the gravitational pull of local minima or pathological attractor states is lessened (62). While this scenario is hypothesized to reflect a therapeutic window of opportunity for the subsequent longer-term de-weighting/flattening of relevant pathological attractors, we are also mindful that the character of the acute state itself may mirror certain states that could be construed as pathological, such as states of dissolved ego-boundaries in early psychosis (62). Thus, we are cautious about depicting psychedelic therapy as a straightforward intervention or *panacea* for all psychological ailments. Such an image would not fairly reflect the complexity and nuance of mental illness, nor indeed, psychedelic therapy, which is a particularly complex intervention that is certainly not without risk.

It is worth noting that despite the large number of studies cited to support this paper's central hypothesis, many were observational in nature or controlled but with small sample sizes, and some lacked control conditions altogether or may have failed to entirely maintain the study blind—a common limitation of psychedelic research. The methodologies also varied widely. Regarding the psychological flexibility construct, caution is advised when consulting studies that report strong correlations with mental health, as they have predominantly applied the AAQ-II. Despite the widespread use of this instrument, a number of recent studies (265, 266) articulated an important critique against it, questioning what the AAQ-II tool is actually measuring; e.g., is it psychological flexibility specifically or a more generic reflection of well-being?

An important limitation of the present paper is that we have not been able to cite direct evidence for the hypothesized prophylaxis *via* psychedelic use or therapy. The most compelling demonstration of prophylaxis would require a longitudinal cohort study that tracks psychedelic “users” and “non-users” across time, while controlling for confounding variables through regression modeling. The above-cited population and prospective observational studies offer the best evidence for the hypothesized prophylaxis, but retrospective sampling is a limitation of the former and self-selecting sample, and attribution biases are a problem with the latter. Our hope is that future large-scale longitudinal cohort studies will include a specific question about psychedelic use.

We are also aware that we have not properly discussed optimal delivery of psychedelic therapy, nor how best to maximize its hypothesized prophylactic potential. Regarding treatment optimization, questions about optimal dosage, frequency of sessions, re-dosing (where indicated), adequate and optimal settings, possible indications and contraindications, which substances work best for what indications and for whom, and the role of age and other characteristics have not been addressed. These matters should be intensively explored in future studies, and this may be done best *via* adaptive or pragmatic trials (267–269).

Recent proposals that psychedelics could be effectively twinned with so-called “third wave” psychotherapies—e.g., mindfulness-based cognitive behavioral therapy and ACT—are also consistent with the proposal that psychedelic therapy can promote psychological flexibility (80, 125, 133, 134, 180, 270). Nevertheless, more research on transdiagnostic targets and processes of change is needed (35). One healthy development would be to find that other psychological approaches and constructs converge in a similar way to those of ACT and psychological flexibility and perhaps share consistent underlying (e.g., neurobiological) mechanisms. Reference to literature on gestalt and psychoanalytic constructs such as raising awareness, creative adjustment, process and field theory (271), defense mechanisms, transference, splitting, projection, abreaction, ego functions and dysfunctions, and the unconscious (272) could help to broaden and enrich our understanding of psychedelic therapy and its mechanisms.

We have proposed that psychedelic therapy represents a promising new therapeutic strategy for treating, enhancing, and maintaining mental health that transcends diagnostic boundaries. We have cited direct evidence for these properties and offered an interdisciplinary “process-of-change” model to explain them. The model is based on a synthesis of theories and observations from psychology and neuroscience, where it is proposed that psychedelics promote an acute state of heightened mind and brain plasticity enabling psychological interventions to work more effectively. We propose that a pharmacologically mediated increase in plasticity provides an opportunity for relaxing and revising canalized habits of mind, brain, and behavior. The present perspective is consistent with the position that mental healthcare and research require a paradigm shift away from solely palliative or reactive “solutions” — e.g., by aiming to treat specific symptoms when they become visible at a critical level. We acknowledge potential risks associated with psychedelic therapy and its integration into mainstream mental healthcare and, thus, emphasize the importance of regulated use, as well as the recognition of the essential role played by context (e.g., therapeutic support) when delivering safe and effective psychedelic therapy.

It is our hope that through a careful roll-out of psychedelic medicine, closely aligned with psychedelic science, we may be able to advance our understanding of how mental illness arises and can be mitigated, or prevented, *via* a particularly deep quality of care. The psychedelic therapy model may be different from previous approaches in mainstream psychiatry in that it recognizes the canalization of thought and/or behavior in response to adversity as a key pathophysiological mechanism and seeks to treat it *via* enhancing plasticity in combination with nurturing support. Thus, psychedelic therapy is a hybrid *drug* × *context* therapeutic model; indeed, it is a combination treatment. It is our hope that a proper recognition of this may help bridge an unnecessary and outdated pharmacology vs. psychology divide in mental healthcare and research (273).

## Data Availability Statement

The original contributions presented in the study are included in the article, further inquiries can be directed to the corresponding author.

## Author Contributions

RK brought the initial idea and wrote the first draft of the manuscript. RC-H wrote sections of the manuscript and contributed substantially to the development of the proposed theoretical model. All authors contributed to the conception, writing, revising the manuscript critically for important intellectual content, and final approval of the manuscript.

## Conflict of Interest

The authors declare that the research was conducted in the absence of any commercial or financial relationships that could be construed as a potential conflict of interest.
